# Semirigid Cantilever Extension System for Splinting Implants: A Clinical Report

**DOI:** 10.1155/2014/192974

**Published:** 2014-08-05

**Authors:** Raissa Micaella Marcello Machado, Luciana de Rezende Pinto, Otacílio Luiz Chagas Júnior, Fernanda Faot

**Affiliations:** ^1^Graduate Program in Dentistry, Prosthodontics Area, School of Dentistry, Federal University of Pelotas (UFPEL), Gonçalves Chaves Street 457, 96015-560 Pelotas, RS, Brazil; ^2^Department of Restorative Dentistry-Prosthodontics Area, School of Dentistry, Federal University of Pelotas-UFPEL, Gonçalves Chaves Street 457, 96015-560 Pelotas, RS, Brazil; ^3^Department of Oral and Maxillofacial Surgery and Maxillofacial Prosthodontics, School of Dentistry, Federal University of Pelotas-UFPEL, Rua Gonçalves Chaves 457, 96015-560 Pelotas, RS, Brazil

## Abstract

In mandibular edentulous patients, treatment based on immediate loading with rigid splinting in the mandible is well accepted; however, it is cost and time dependent, which sometimes limits this type of rehabilitation. To overcome these problems, the technique of immediate loading using a semirigid splinting extension system has been developed. Its advantages include low cost, technical feasibility, and reduced clinic time. This clinical report presents the applicability and the predictability of semirigid splinting of implants in the mandibular arch of an edentulous patient using a *distal extension bar prosthesis system.*

## 1. Introduction

With advances in dental implant geometry and surface texture, prosthetic connections, and simplified surgical techniques, the concept of immediate and early loading has gained credibility and predictability. However, the main guiding factors in the success of implant-supported prostheses in the edentulous mandible are the implant primary stability and the need for splinting the implants, through a rigid metal infrastructure, to prevent micromotion of the implants and provide ideal conditions for osseointegration [1].

However, the laboratory logistics required to implement the standard protocol, the financial costs involved in this type of rehabilitation, and the need for rapid processing [2] limit the scope of rehabilitative modality, for both the professional and the patient. In order to remedy these difficulties, prefabricated bars have been proposed for use with* hybrid prostheses* [3]. This alternative embodies an implant-supported mandibular prosthesis that has enabled the application of immediate or early loading, as shown by a system of semirigid splinting composed of prefabricated metallic distal extensions with the addition of conventional acrylic resin, called* distal extension bar prosthesis system *(DEBPS) [4].

Despite the use of semirigid splinting, no studies in the literature—even those discussing fabrication tips and predictability of this treatment modality—indicate the maximum time that such splinting can be used. Only one study conducted by Lee et al. [4] used this system in a sample of fifteen edentulous patients with only 8-month follow-up. Based on this clinical study, the only specific prerequisite for the adoption of this technique is the need to obtain primary stability during implant installation if the immediate or early loading is planned or desired [4–6]. This clinical report describes the predictability of semirigid implant splinting in the rehabilitation of an edentulous mandible by means of a DEBP, with an 18-month follow-up.

## 2. Case Report

**Figure 1:**
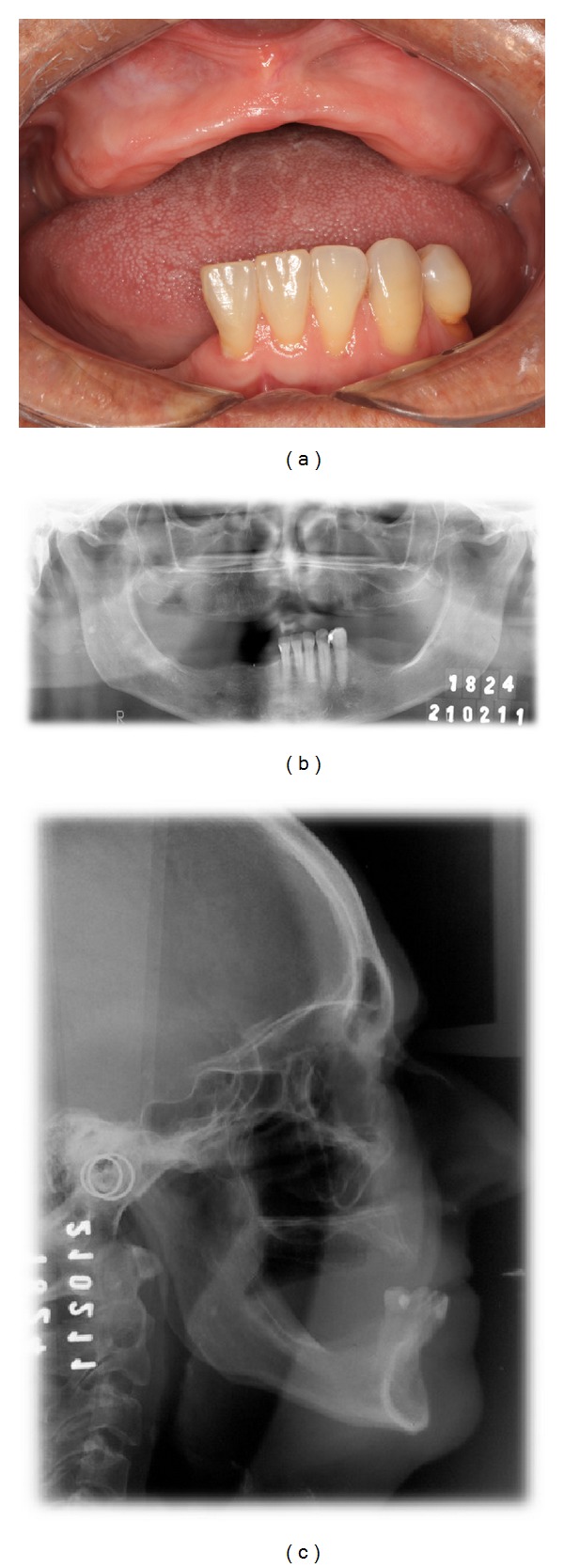
(a) Clinical exam: edentulous maxillary and partially edentulous mandible. (b) Radiographic exam: panoramic radiography. Mandibular height favoring dental installation implants. (c) Radiographic exam: teleradiography. Favorable maxillomandibular relationship to the installation of dental implants and prosthetic rehabilitation treatment.

Patient V.E.C., 61-year-old female in a good general health was referred to the Dental School - Prosthodontics Unit of the Federal University of Pelotas for maxillary and mandibular oral rehabilitation. During the prosthesis evaluation it was observed a maxillary complete denture in fair condition however, presenting a good retention, stability and support. In the mandibular arch, the patient had a removable partial denture without retention or stability, which was the patient's main complaints, resulting in difficulties in chewing, in speech, and in her social life.  Table 1 presents the treatment steps that were taken throughout the patient rehabilitation. Following the interview, clinical (Figure 1(a)) and radiographic examinations were performed (Figures 1(b)-1(c)). It was observed that the level of posterior mandibular alveolar bone absorption would allow only the use of short implants, requiring an unfavorable crown/implant ratio. Therefore, it was decided on the installation of 5 dental implants (Titamax Cortical with Morse Taper, 3.75 × 13 mm, Neodent Osseointegrated Implants, Curitiba, PR, Brazil) in the interforaminal region of the mandible with the use of DEBPS, mainly because of the economical limitations of the patient.

**Figure 2:**
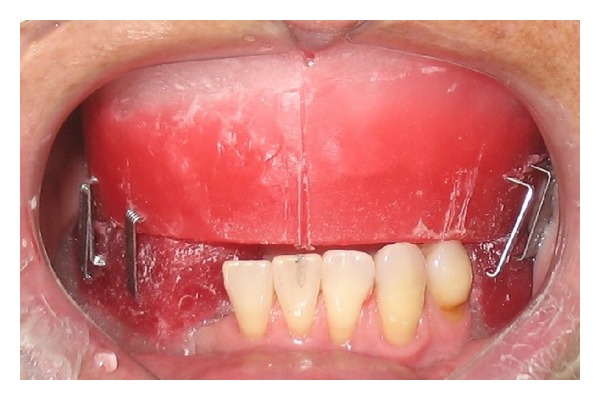
Evaluation of the reestablished VDO.

**Figure 3:**
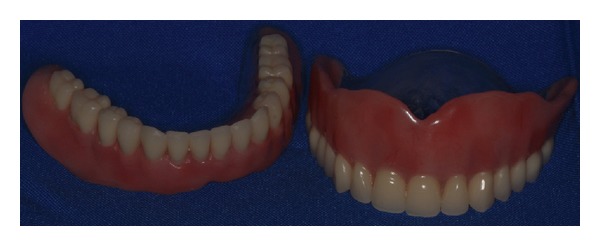
New complete dentures.

**Figure 4:**
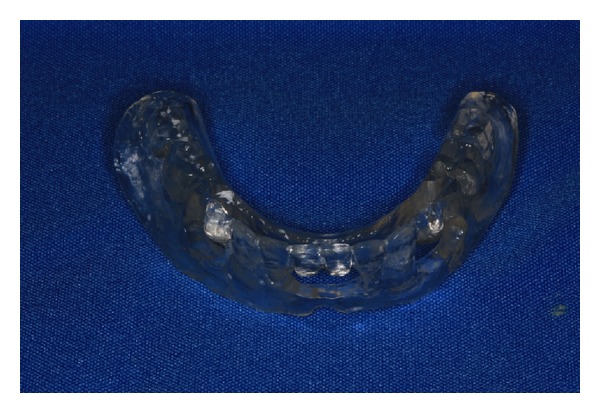
Surgical guide.

**Figure 5:**
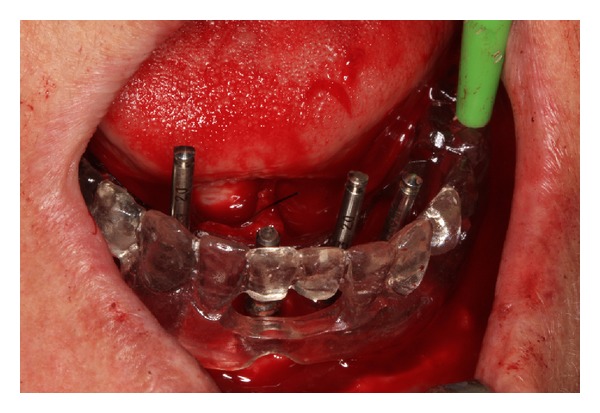
Implant placement.

**Figure 6:**
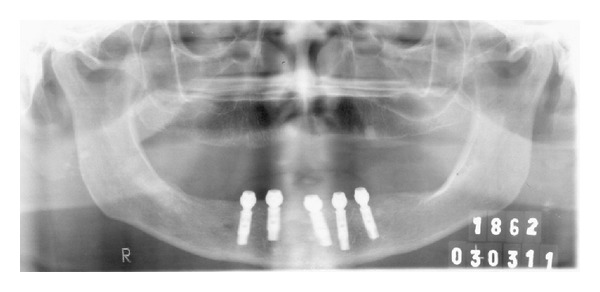
Panoramic radiography after implants placement.

**Figure 7:**
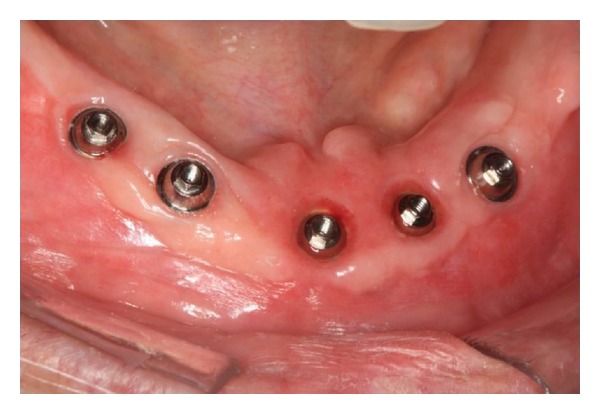
Prosthetic abutments and peri-implant tissue after the 15th day.

**Figure 8:**
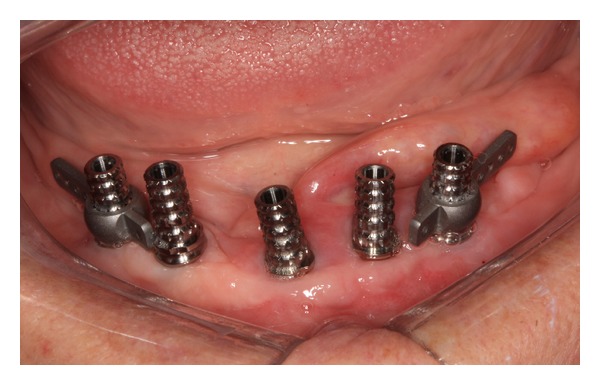
Installation of titanium cylinders and distal bars of cantilever extension system (implants 1 and 5).

The presurgical phase involved all the steps required to perform a complete denture with especial attention to the maxillomandibular relation record with a correct establishment of the vertical dimension of occlusion (Figures 2 and 3). The remaining teeth were extracted (no. 21, no. 22, no. 23, no. 24, and no. 25) and multifunctional surgical guide (Figure 4) was used during the drilling sequence, guiding the osteoplasty into correct parallel positioning of the implants (Figure 5). An insertion torque of up to 40 N*·*cm guaranteed the primary implant stability. Prosthetic abutments (CM Mini Conical Abutment, Neodent) were installed and a postsurgical panoramic radiography was performed (Figure 6). Prosthetic procedures began 15 days after the surgery 6 (Figure 7) with the installation of one semi-rigid cantilever extension system with titanium bars placed in the 2 distal abutment cylinders (Figure 8). The choice of waiting for this healing time of 2 weeks was to promote better soft tissue positioning and the reposition of the floor of the mouth, which presented a high muscle insertion. These anatomical conditions did not permit installing the DEBPS at the time of the surgery. However, the immediate loading was not discarded. According to Esposito et al. [7], 3 loading protocols are well established in the literature and are dependent of implants primary stability and the bone properties (quality and quantity): immediate (within 1 week); early (from 1 week to 2 months); or conventional (after 2 months). A high value of insertion torque (at least 35 Ncm) seems to be one of the prerequisites for a successful immediate/loading procedure [7]. If resonance frequency analysis was used to assess the primary stability, an ISQ value of at least 60 should be obtained [8]. Conventional loading is recommended in the following situations: (i) primary implant stability could not be achieved, (ii) type IV bone, (iii) patient with parafunctional habits as bruxism or clenching, (iv) alveolar ridge augmentation procedures and (v) compromised bone as observed in osteoporosis and diabetes.

**Figure 9:**
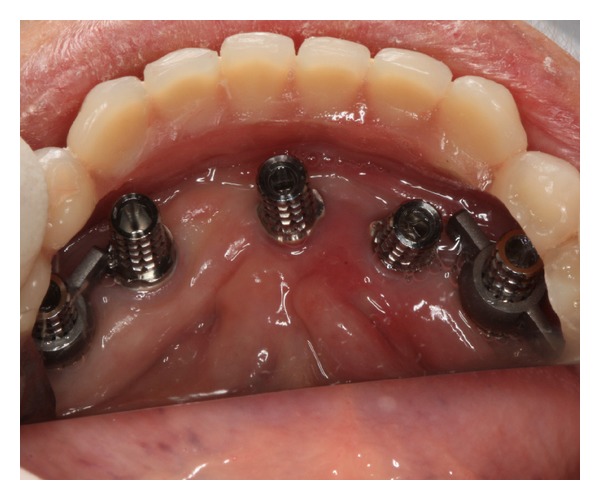
Positioning of the lower denture with titanium cylinders and distal bar.

**Figure 10:**
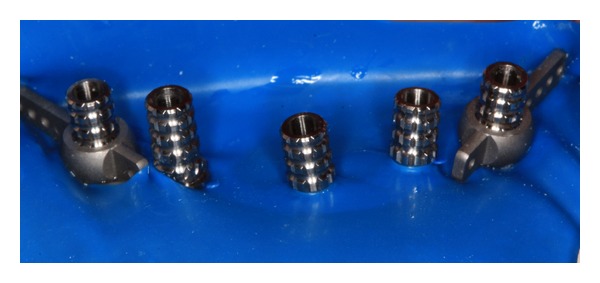
System protection with rubber dam to capture the titanium cylinders and distal bar.

**Figure 11:**
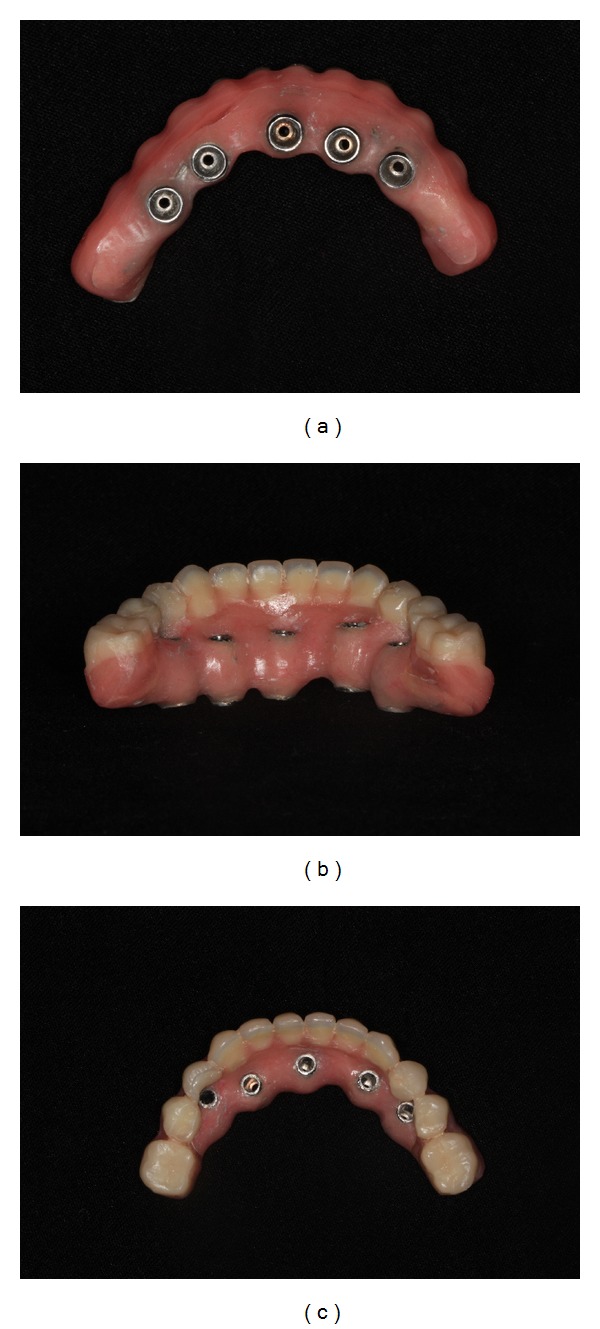
Mandibular denture after capturing the titanium cylinder and distal bar.

**Figure 12:**
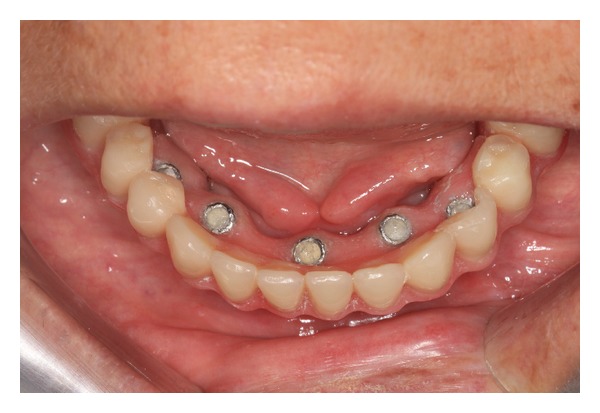
Screwed mandibular denture and its relation to the tissues of the buccal floor.

**Figure 13:**
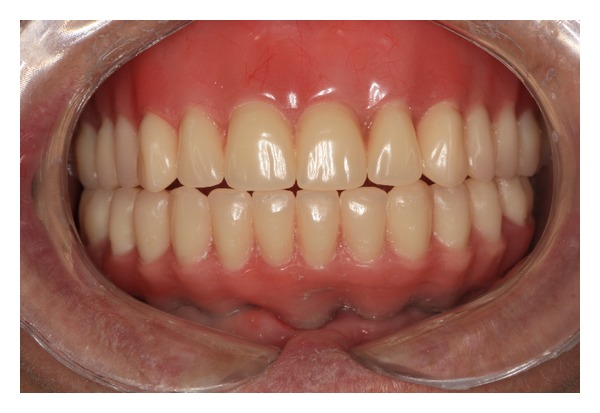
Complete dentures in occlusion.

**Figure 14:**
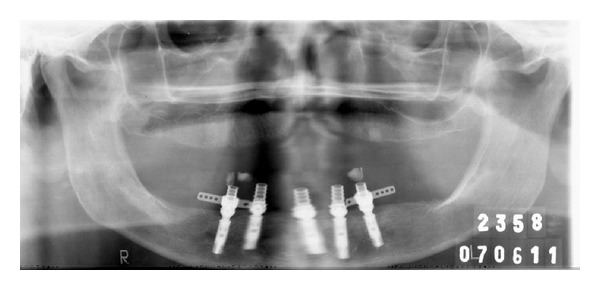
Panoramic radiography after 7 days of the prosthesis installation.

**Figure 15:**
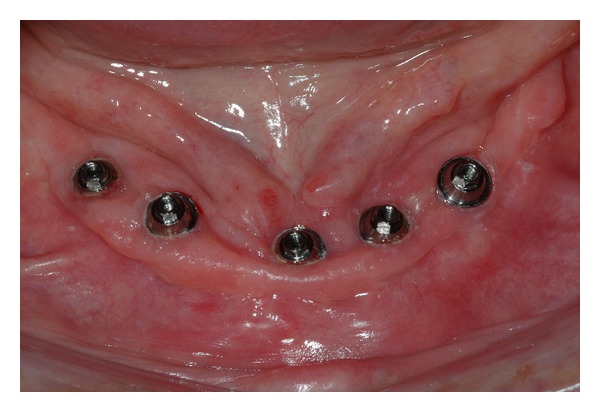
Clinical aspect of the peri-implant tissue after 2 years of installation.

**Figure 16:**
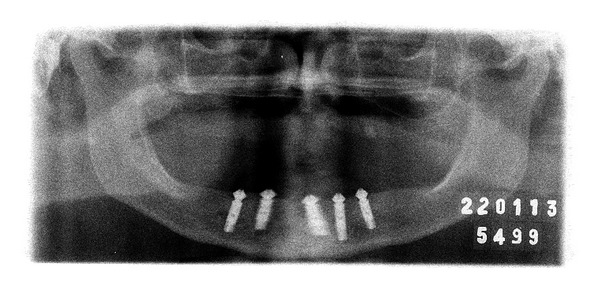
Radiographic control at 2-year follow-up.

The lingual surface of the mandibular prosthesis corresponding to the location of each cylinder was adjusted (Figure 9). To isolate the field and prevent the flow of acrylic resin over the prosthetic abutment and peri-implant mucosa, a rubber dam was adapted over the cylinders (Figure 10). After the prosthesis was repositioned in the mouth, the distal extensions were fixed to the cylinders with acrylic resin (New Truliner, Bosworth Company, Ill, USA). The length of the distal cantilever in the mandibular implant-supported prosthesis was reduced by the elimination of the distal portion from the first molar. Mandibular denture after capturing the titanium cylinder and distal bar had hygienic pontics carved between the implants (Figures 11(a), 11(b), and 11(c)) to promote better hygiene of the peri-implant soft tissue and occlusal adjustments were performed (Figures 12 and 13). After 7 days, the patient underwent radiography for the final clinical evaluation (Figure 14), and follow-ups were performed every 3 months. After 2 years of monitoring, the mandibular implant-supported prosthesis and implants were reassessed to the plaque accumulation and marginal mucosal conditions according to Mombelli et al. [9] (Table 2). Plaque accumulation around implants could be seen by the naked eye (score 2) (Figure 15). With all the implants, no occurrence of gingival inflammation around the implants was reported (score 0) nor was there a need for denture repairs due to broken teeth or resin. The integrity of the peri-implant bone was also verified by radiographic examination (Figure 16).

## 3. Discussion

There are several benefits of immediate or early loading by semirigid splinting, for example, eliminating the need for maintaining or replacing a removable prosthesis that may cause patient discomfort or postoperative pain, slow the healing process, or cause premature exposure of the implants. Repeated injuries due to lack of retention or stability could ultimately result in an increased number of visits required for maintaining the prosthesis. Emotional, aesthetic, and functional benefits are also promoted for the patient who would otherwise be toothless.

Improving healing facilitates the formation of soft tissue [3, 4]. In this case, the option of semirigid splinting was adopted to promote more rapid peri-implant tissue healing and to reposition the floor of the mouth, which had a high muscle insertion. Semirigid splinting systems also enable the use of small accommodation fasteners, which are feasible because of the beneficial micromotion permitted by the absence of metal infrastructures [3, 4, 10].

Also, reducing the cost of rehabilitation, even temporarily, makes it available to more people and improves life quality through the restoration of oral function. Another consideration is that this prosthesis might provisionally serve the patient until he or she has the financial resources to subsequently undergo another form of rehabilitation. In addition, an implant-fixed prosthesis without the cast rigid framework could be an option in cases when there is enough time or technical knowledge to fabricate a cast framework within the period recommended for immediate or early loading, this technique would eliminate a gap in treatment options and, at the same time, open the doors to a greater number of practitioners.

However, in making a decision to use immediate loading with associated semi-rigid splinting, each edentulous patient should be evaluated preoperatively to ensure that he or she meets the clinical criteria that include adequate bone quality (types I, II, or III), sufficient bone height and width, and the ability to achieve adequate anteroposterior distribution during cantilever fabrication [10]. In cases in which the patient already has a complete denture under conditions acceptable for rehabilitation, it is recommended that, due to the coupling between cylinders and bars held only in the acrylic resin, the technique is restricted to extremely reabsorbed ridges, so that the added thickness of material can supply more rigidity and resistance to the assembly.

## 4. Conclusion

The use of the semirigid distal cantilever extension system for splinting implants is a viable treatment option for rehabilitating edentulous mandibles that meet strict criteria for immediate or early loading.

## Figures and Tables

**Table 1:** Treatment steps.

Steps	Performed treatments	Figure
1	Initial phase: patient examination and diagnosis	Figures 1(a), 1(b), and 1(c)
2	Presurgical phase: assessment of the vertical dimension of occlusion (VDO), evaluation of the reestablished VDO, diagnostic waxing, and new complete denture	Figures 2 and 3
3	Surgical guide based on maxillomandibular relationship	Figure 4
4	Teeth extraction (no. 21, no. 22, no. 23, no. 24, and no. 25) and implant placement	Figure 5
5	Postsurgical radiography	Figure 6
6	Abutment placement and peri-implant tissue	Figure 7
7	Installation and capture of titanium cylinders and distal bars	Figures 8, 9, and 10
8	Mandibular implant-supported prosthesis installation	Figures 11, 12, and 13
9	Follow-ups every 3 months and maintenance routine of the implants	Figures 14, 15, and 16

**Table 2:** Indices used to assess plaque accumulation and marginal mucosal conditions around oral implants according to Mombelli et al. [9].

Indices to assess plaque accumulation	
(0) No detection of plaque	
(1) Plaque only recognized by running a probe across the smooth marginal surface of the implant	
(2) Plaque can be seen by the naked eye	
(3) Abundance of soft matter	

Indices to assess marginal mucosal conditions	

(0) No bleeding when a periodontal probe is passed along the mucosal margin adjacent to the implant	
(1) Isolated bleeding spots visible	
(2) Blood forms a confluent red line on mucosal margin	
(3) Heavy or profuse bleeding	
